# High abundance of *Stenotrophomonas maltophilia* in slaty-backed gull breeding in Northern Japan

**DOI:** 10.1128/spectrum.00947-26

**Published:** 2026-07-06

**Authors:** Mako Kawai, Ren Fujita, Riko Ohshima, Takehiko Kenzaka

**Affiliations:** 1 Faculty of Pharmaceutical Sciences, Himeji Dokkyo University 70299, Himeji, Hyogo, Japan; 2 Faculty of Science and Engineering, Setsunan University 47731, Neyagawa, Osaka, Japan; Connecticut Agricultural Experiment Station, New Haven, Connecticut, USA

**Keywords:** *Stenotrophomonas maltophilia*, antibiotic resistance, gull, migratory bird, avian

## Abstract

*Stenotrophomonas maltophilia* is a gram-negative bacillus that is widely distributed in the natural environment and is known to cause opportunistic infections. While it has been isolated from diverse wildlife, the relationship between migratory birds and this bacterium is poorly understood. In this study, we investigated fecal samples from migratory slaty-backed gulls (*Larus schistisagus*) breeding in Northern Japan by 16S rRNA sequencing and examined the characteristics of multidrug-resistant bacteria. We found that *S. maltophilia* represented 41% of all bacterial 16S rRNA gene sequences. *S. maltophilia* capable of growing on agar medium supplemented with multiple antibiotics was present at levels up to 10⁴ CFU/g, and most of the 60 isolates exhibited strong biofilm-forming ability and swimming motility. Although trimethoprim–sulfamethoxazole and minocycline were relatively effective, 18% of the isolates showed resistance to both trimethoprim–sulfamethoxazole and levofloxacin, which are widely used as therapeutic drugs. All strains carried genes involved in multidrug efflux pumps and aminoglycoside resistance. The frequencies of virulence-associated determinants were as follows: *stmPr1* (major extracellular protease), 56.7%, and *smf-1* (type 1 fimbriae, biofilm-related gene), 100%. These findings suggest that migratory gulls play a role in the dissemination of antibiotic-resistant *S. maltophilia* in natural environments.

**IMPORTANCE:**

*Stenotrophomonas maltophilia* has emerged as an opportunistic pathogen that causes pneumonia and bloodstream infections. Despite its clinical importance, there are few reports describing its association with migratory birds. This study revealed that the intestines of slaty-backed gulls harbor a high proportion of this bacterium. Among the multidrug-resistant bacteria examined, all isolates were identified as *S. maltophilia*. While most isolates were susceptible to commonly used antimicrobial agents such as trimethoprim–sulfamethoxazole, minocycline, and levofloxacin, some exhibited resistance to these antibiotics. Furthermore, all isolates showed strong biofilm-forming capacity and swimming motility, similar to clinical isolates, thereby possessing the potential to adhere to and develop biofilms on artificial surfaces such as medical devices, as well as on mucosal surfaces. Collectively, these findings indicate that migratory gulls may carry and transport antibiotic-resistant *S. maltophilia*.

## OBSERVATION

Recent studies have revealed that migratory birds transport antibiotic-resistant bacteria (ARB) across borders (1, 2). Although the birds themselves are not administered antibiotics, waterfowl such as gulls and ducks often feed near urban wastewater treatment plants, landfills, or rivers and farmlands that receive livestock waste, which contain antibiotics and ARB from humans or livestock (3). A key feature of migratory birds is their ability to travel thousands of kilometers, carrying pathogens acquired in one country to another country or continent while still harboring them in their intestines, spreading them through feces (4–7). This poses a risk of introducing ARB to areas where antibiotic use is rare. Typical ARBs detected in migratory birds include extended-spectrum beta-lactamase-producing *Escherichia coli*, colistin-resistant *Enterobacteriaceae*, *Salmonella* spp., and *Campylobacter* spp. (8–12).

Slaty-backed gulls (*Larus schistisagus*) breed in Northern Japan during the summer and travel an average of 25 km per day within their habitat to forage, although some individuals may travel up to 100 km (13). In winter, they migrate from East Asia to Southeast Asia, covering distances of over 4,000 km. Previous studies have shown that slaty-backed gulls, like gulls in Europe and South America, frequently harbor colistin-resistant *E. coli* (12). In this study, we screened multidrug-resistant bacteria in slaty-backed gulls and investigated their types and genetic characteristics to better understand their resistance profiles.

A total of 19 fecal specimens of *L. schistisagus* were collected from three breeding areas in Akkeshi in Hokkaido, Japan, in September 2024. Only fresh feces were collected in the absence of other birds. The abundance of total bacteria and *Stenotrophomonas maltophilia* in the feces is shown in Table 1. The total bacterial load in the feces was on the order of 10^7^–10^8^ cells/g, and *S. maltophilia* accounted for 14%–47% of all bacterial 16S rRNA gene sequences (median, 41%), which was higher than previously reported for aquatic environments (14). Culturable bacteria were present at approximately 10^4^–10^6^ CFU/g, corresponding to about 0.01%–1.4% of the total bacterial population. All resistant bacteria capable of growing on R2A agar supplemented with meropenem, ciprofloxacin, and colistin were identified as *S. maltophilia* strains. These strains were detected in 53% of the samples at levels of approximately 10^2^−10^4^ CFU/g, accounting for <0.01%–3.4% (median, 0.10%) of the total viable bacterial count.

**TABLE 1:** Abundance of bacteria and *S. maltophilia* in the feces of *Larus schistisagus* (*n* = 19)

	Total direct count*^a^*	Total viable count*^b^*		16S rRNA *S. maltophilia*^*c*^	Resistant viable *S. maltophilia^d^*	
	(cells/g)	(CFU/g)	(%)	(%)	(CFU/g)	(%)
Range	3.5 × 10^7^ to 4.3 × 10^8^	1.3 × 10^4^ to 1.5 × 10^6^	0.01−1.4	14−47	<4.4 × 10^2^ to 2.1 × 10^4^	<0.01−3.4
Median	2.1 × 10^8^	2.1 × 10^8^	0.22	41	5.0 × 10^2^	0.10

^*a*^ Fecal samples were stained by SYBR Green I.

^*b*^ Fecal samples were cultured on R2A, and % indicates the percentage of total direct count.

^*c*^ 16S amplicon sequencing was performed and shown as a percentage of total sequence reads.

^*d*^ Fecal samples were incubated on R2A containing meropenem, ciprofloxacin, colistin (or amikacin), and cycloheximide, and isolates were confirmed to be *S. maltophilia* by real-time PCR. % indicates the percentage of total viable count.

As indicators of antimicrobial susceptibility, the MIC range, MIC50, and MIC90 in the 60 isolates are shown in Table 2 and Table S1. For *S. maltophilia*, the human clinical breakpoints for resistance to trimethoprim–sulfamethoxazole, minocycline, and levofloxacin were defined as ≥4/76, ≥4, and ≥8 μg/mL, respectively (15). Trimethoprim–sulfamethoxazole showed resistance in a subset of isolates. Minocycline exhibited relatively low MICs, suggesting that many isolates were susceptible to minocycline, whereas susceptibility to tetracycline was low. Levofloxacin showed considerable variability in susceptibility among isolates, and some isolates were not inhibited even at high concentrations. Eighteen percent of the isolates (11/60) showed resistance to both trimethoprim–sulfamethoxazole and levofloxacin. These antibiotics are used clinically to treat infections caused by this species. Breakpoints for the other antibiotics tested in this study have not been established. *S. maltophilia is* intrinsically resistant to multiple antimicrobial agents, such as meropenem, amikacin, and gentamicin, and readily develops multidrug resistance (16). Most isolates in our study demonstrated reduced susceptibility to ciprofloxacin and colistin (Table S1). This aligns with trends in clinical isolates, involving multidrug efflux pumps, target-site alterations, and other mechanisms (17).

**TABLE 2:** Characteristics of the 60 *S. maltophilia* isolates

Characteristics	Values
Antibiotic susceptibility*^a^*	
Trimethoprim–sulfamethoxazole (1:19)	0.125/2.375–32/608, 0.5/9.5, 4/76
Minocycline	0.5–4, 1, 2
Levofloxacin	1–64, 6, 16
Biofilm*^b^*	
Strong	59 (98.3%)
Moderate	1 (1.5%)
Swimming*^b^*	
Strong	56 (93.3%)
Moderate	4 (6.7%)
Swarming*^b^*	
Negative	60 (100%)
Virulence, biofilm, and antibiotic resistance gene*^b^*	
*stmpr1*	35 (58.3%)
*stmpr2*	0 (0%)
*smf-1*	60 (100%)
*aph3*	60 (100%)
*smeABC*	60 (100%)

^*a*^ MIC range, MIC_50_, MIC_90_ (µg/mL).

^*b*^ Number of positive colonies in 60 isolates (%). The presence of virulence genes was examined via real-time PCR using specific primers shown in Table S3.

Regarding biofilm formation, almost all isolates (59/60) exhibited strong biofilm formation, and the remaining isolate showed moderate biofilm formation (Table 2 and Table S1). The isolates exhibited high swimming motility but did not display swarming motility on solid surfaces, which is a representative phenotypic characteristic of this species.

Genomic analysis identified isolate C5 as ST714 and isolate E2 as a novel sequence type, ST1482 (Table S2). While ST714 was previously reported in a clinical isolate from Northern Thailand (18), neither of our isolates matched the approximately 90 *S. maltophilia* strains from Japan currently registered in the MLST database, which encompass human, animal, and environmental sources.

Major virulence genes (*stmpr1*, *stmpr2*, and *smf-1*), biofilm genes (*rmlA*, *spgM*, and *rpfF*), and antibiotic resistance genes [*aph(3′)IIc*, *blaL1*, and *smeABC*] listed in previous studies (19, 20) were detected in the strains (Table S2). Regarding some genes, the proportions of PCR-positive isolates were examined, and *smf-1*, *aph(3′)IIc*, and *smeABC* were detected in all isolates (Table 2 and Table S1). The type 1 fimbriae, biofilm-related gene *smf-1*, encodes a factor involved in host attachment and biofilm formation. The presence of *smf-1* is consistent with the strong biofilm-forming ability described above and has been implicated in biofilm formation on cystic fibrosis-related epithelial cells (21). The *aph(3′)IIc* gene encodes an aminoglycoside-modifying enzyme (phosphotransferase) and contributes to resistance to aminoglycosides such as amikacin and gentamicin. The *smeABC* operon is known as an RND-type multidrug efflux pump and represents a major determinant of multidrug resistance. These results suggest that this species possesses a genetic basis for resistance and survival against various antimicrobial agents in environmental and host-associated settings, including antibiotics, heavy metals, and disinfectants (22). StmPr1 and StmPr2 function as extracellular proteases and are involved in host tissue destruction and cytotoxic effects (21). *StmPr1* was detected in approximately half of the isolates (58.3%). Previous studies have reported that the prevalence of these virulence factors differs among clinical isolates (19, 22).

Given their biofilm-forming ability, motility, and the prevalence of multidrug efflux pumps, these isolates are likely well adapted for survival and persistence in aquatic environments and drainage systems. Furthermore, they may have the potential to adhere to artificial surfaces and mucosal tissues, although this requires experimental verification. Although these *S. maltophilia* isolates were obtained from wild animals, they exhibited resistant profiles comparable to those observed in recent clinical isolates from Europe, Asia, and Latin America (23). Because therapeutic options are restricted to only a few agents, such as trimethoprim–sulfamethoxazole and minocycline, these strains would pose a significant clinical challenge if they were to invade humans. Such transmission could potentially occur through environmental contamination, particularly via gull feces deposited in water sources, on surfaces, or in other shared environments.

There have been few reported associations between migratory birds and *S. maltophilia* (24, 25). In our study, fecal samples from *L. schistisagus* contained approximately 10^2^–10^4^ CFU/g of *S. maltophilia* capable of growing on R2A medium supplemented with multiple antibiotics. In contrast, the numbers of viable *S. maltophilia* were reported to be several tens of CFU per milliliter for treated wastewater, pond water, and river water (14). Our study suggests that intestinal *S. maltophilia* in *L. schistisagus* likely behaves as a normal commensal or environmental symbiont for the gulls themselves. Gulls frequently inhabit areas in close proximity to human activities, including coastal areas, harbors, reclaimed land, fishing ports, and urban waste disposal sites. Their feces are widely distributed across aquatic environments (such as seas, rivers, and ports), soil, and concrete surfaces. Indeed, *S. maltophilia* has been isolated from diverse water-associated habitats, ranging from artificial infrastructure (e.g., hospital plumbing systems and municipal sewage) to natural ecosystems (e.g., rivers, lakes, and agricultural soils), as well as from a variety of host organisms (17). The findings of this study underscore the significant role of migratory gulls as both an environmental reservoir and a vector for the spread of this bacterium.

However, given the small sample size from a limited geographic area in this pilot study, further large-scale investigations are needed to confirm the extent to which these migratory birds carry such ARB. In humans, *S. maltophilia* is a well-known opportunistic pathogen, particularly affecting immunocompromised and hospitalized patients. In the future, comparative genomic analyses of environmental, wildlife, and clinical isolates may allow us to trace the origins of antibiotic-resistant strains and the transmission routes among humans, the environment, and wild birds.

## Data Availability

16S rRNA amplicon sequencing data are available through the National Center for Biotechnology Information (NCBI) via BioProject PRJNA1438084. Genome sequence data are available through the NCBI via BioProjects PRJNA1438172 and PRJNA1438154. All other data supporting the findings of this study are included in this article and its Supplemental materials.

## SUPPLEMENTAL MATERIAL

Supplemental materials spectrum.00947-26-s0001.pdf Supplemental Materials and Methods and Tables S1 to S3.

ASM does not own the copyrights to Supplemental Material that may be linked to, or accessed through, an article. The authors have granted ASM a non-exclusive, world-wide license to publish the Supplemental Material files. Please contact the corresponding author directly for reuse.
