# Supplementary material for: High abundance of Stenotrophomonas maltophilia in slaty-backed gull breeding in Northern Japan
Source: Microbiol Spectr. 2026 Jul 6;14(8):e00947-26. doi: 10.1128/spectrum.00947-26 (PMC13435717; doi:10.1128/spectrum.00947-26)
Supplement: Supplementary file 1 [file spectrum.00947-26-s0001.pdf]

Supplemental information

Materials and Methods

Environmental samples. A total of 19 fecal specimens were collected from three breeding areas on Akkeshi in Hokkaido, Japan, in September 2024. Only fresh feces were collected in the absence of other birds.

Determination of bacterial counts, and isolation of multidrug resistant bacteria. Total direct counts in fecal samples were determined by staining with SYBR Green I (Thermo Fisher Scientific Inc., Waltham, MA, USA). The number of culturable bacteria in the fecal samples was determined by plate counting on R2A agar after incubation at 37°C for 1 week under aerobic conditions. A total of 135 colonies, including four to seven multidrug-resistant colonies per fecal sample, were isolated on R2A agar supplemented with 4 µg/mL meropenem, 2 µg/mL ciprofloxacin, 10 µg/mL colistin, and 50 µg/mL cycloheximide to suppress fungal growth, after incubation at 37°C for 2 days under aerobic conditions. After single-colony purification, all bacterial isolates were identified as *S. maltophilia* by real-time PCR using the Smalto-For and Smalto-Rev primers and the Smalto-Probe (1). Sixty colonies were subjected to further characterization.

Antibiotic susceptibility testing. The minimum inhibitory concentration (MIC) was determined using the agar dilution method (2).

Biofilm-forming ability. The biofilm-forming ability of the isolates was assessed using a crystal violet staining assay (3). Bacterial cultures (1.0 McFarland standard, diluted 1:100) were incubated in microtiter plates at 37°C for 24 h. After washing and fixing, the biofilms were stained, destained, and quantified by measuring the optical density (OD) at 585 nm. A cut-off OD value (ODc) was defined as three standard deviations (SD) above the mean OD of the negative control wells. Based on this ODc, the strains were classified into four categories: non-biofilm producers ($OD \leq ODc$), weak biofilm producers ($ODc < OD \leq 2 \times ODc$), moderate biofilm producers ($2 \times ODc < OD \leq 4 \times ODc$), and strong biofilm producers ($OD > 4 \times ODc$) (4).

Motility Assay. The swimming motility of *S. maltophilia* was assessed on Luria-Bertani (LB) agar plates (10 g/L tryptone, 5 g/L yeast extract, 5 g/L NaCl, and 3 g/L agar) as previously described by Rajeev et al. (5). A 5-µL aliquot of the bacterial culture (0.5 McFarland standard) was inoculated at the center of each plate and incubated at 37°C

for 24 h. Based on the measured halo diameters, swimming motility was categorized as high (>10 mm), moderate (>7 to ≤10 mm), or low (>6 to ≤7 mm) [Cruz-Córdova et al., 2020]. Swarming motility was similarly evaluated using LB agar plates containing a higher agar concentration (5 g/L).

Genomic sequencing and analyses. The isolated strains C5 and E2 were grown on LB agar at 37°C for 1 day, and genomic DNA was extracted using the QIAamp DNA Mini Kit (Qiagen, Germany). A sequencing library was prepared with the SQK-NBD114.24 Native Barcoding Kit 24 V14 (Oxford Nanopore Technologies, Oxford, UK) according to the protocols. Sequencing was performed on a MinION device using an R10.4.1 Flongle Flow Cell (FLO-FLG114) for 24 h with super-accurate basecalling (v4.3.0). A total of 46,476 for C5 and 16,670 reads for E2 (470,619,303 and 128,704,932 base, respectively) with a minimum quality threshold of Q8 (N50, 14,148 and 14,269 base, respectively) were assembled with Flye v2.9.6 (6) and polished with Medaka v2.1.1 (https://github.com/nanoporetech/medaka). The genome was annotated using Prokka v1.14.6 (7). Assembly quality (completeness and contamination) and average nucleotide Identity were assessed using CheckM v1.2.4 (8) and FastANI v1.3 (9), respectively. The nucleotide sequences of seven housekeeping genes were compared to the reference sequences on the PubMLST database (https://pubmlst.org/smaltophilia/: accessed on 21 May 2026) for identifying the allelic number of each locus and the classifying STs. The new sequence types of *S. maltophilia* in the present study were deposited in PubMLST: https://pubmlst.org/organisms/stenotrophomonas-maltophilia. Antibiotic resistant genes were identified using ResFinder v4.7.2 with thresholds of 80% identity and 60% minimum length (10), and the Comprehensive Antibiotic Resistance Database v4.0.1 with strict-hit criteria (11).

Detection virulence and antibiotic resistance genes. The presence of virulence genes (*stmpr1*, *Stmpr2*, and *smf-1*) and antibiotic resistance genes (*smeABC* and *aph(3')IIc*) was examined via SYBR Green real-time PCR using specific primers shown in Table S3 (12, 13). The PCR mixtures were prepared using the PowerUp™ SYBR™ Green Master Mix (Thermo Fisher Scientific Inc.). Amplification was performed on a Thermal Cycler Dice® Real Time System III (TaKaRa Bio Inc., Kusatsu, Japan) with the following thermal cycling conditions: initial incubation at 50°C for 2 min and 95°C for 2 min, followed by 40 cycles of 95°C for 15 s, 54°C for 30 s, and 72°C for 1 to 2 min. A final melting curve analysis was conducted by ramping the temperature from 72°C to 95°C.

16S rRNA profiling. DNA was extracted from approximately 100 mg of fecal samples using the QIAamp Fast DNA Stool Mini Kit (Qiagen KK, Tokyo, Japan). Full-length 16S rRNA amplicon sequencing was performed on the MinION platform (Oxford Nanopore Technologies, Oxford, UK) equipped with an R10.4.1 Flongle flow cell (Oxford Nanopore Technologies). Sequencing libraries were prepared from 10 μL of the extracted DNA using the 16S Barcoding Kit 1-24 (Oxford Nanopore Technologies) according to the manufacturer's instructions. The resulting FASTQ files were analyzed using the cloud-based EPI2ME wf-16S v1.5.0 (Oxford Nanopore Technologies), and total 191,990 reads with a quality score of ≥9 were retained for downstream taxonomic classification.

References

1. Fraser TA, Bell MG, Harris PNA, Bell SC, Bergh H, Nguyen TK, Kidd TJ, Nimmo GR, Sarovich DS, Price EP. 2019. Quantitative real-time PCR assay for the rapid identification of the intrinsically multidrug-resistant bacterial pathogen *Stenotrophomonas maltophilia*. Microb Genom. 5:e000307. DOI: 10.1099/mgen.0.000307.
2. Lewis II JS. 2025. CLSI M100-Ed35 Performance Standards for Antimicrobial Susceptibility Testing Standards. 35th Edition. Clinical and Laboratory Standards Institute.
3. Di Bonaventura G, Spedicato I, Antonio D, Robuffo I, Piccolomini R. 2004. Biofilm formation by Stenotrophomonas maltophilia: Modulation by quinolones, trimethoprim-sulfamethoxazole, and ceftazidime. Antimicrob Agents Chemother. 48:151-160.
4. Stepanović S, Vuković D, Hola V, Di Bonaventura G, Djukić S, Cirković I, Ruzicka F. 2007. Quantification of biofilm in microtiter plates: overview of testing conditions and practical recommendations for assessment of biofilm production by staphylococci. APMIS. 115:891-899. DOI: 10.1111/j.1600-0463.2007.apm_630.x.
5. Rajeev R, Kannan P, Sundaram S, Mohan SB, Radjendirane S, Harnathbhai CJ, Subbaiyan A, Naveenkumar V, Mohanadasse NQ, Savariraj WR, et al. 2025. First Report of Stenotrophomonas maltophilia from Canine Dermatological Infections: Unravelling Its Antimicrobial Resistance, Biofilm Formation, and Virulence Traits. Antibiotics. 14:639. DOI: 10.3390/antibiotics14070639.

6. Lin Y, Yuan J, Kolmogorov M, Shen MW, Chaisson M, Pevzner PA. 2016. Assembly of long error-prone reads using de Bruijn graphs. Proc Natl Acad Sci U S A. 113(52):E8396-E8405. DOI: 10.1073/pnas.1604560113.
7. Seemann T. 2014. Prokka: rapid prokaryotic genome annotation. Bioinformatics. 30(14):2068-2069. DOI: 10.1093/bioinformatics/btu153.
8. Parks DH, Imelfort M, Skennerton CT, Hugenholtz P, Tyson GW. 2015. CheckM: assessing the quality of microbial genomes recovered from isolates, single cells, and metagenomes. Genome Res. 25(7):1043-1055. DOI: 10.1101/gr.186072.114.
9. Jain C, Rodriguez-R LM, Phillippy AM, Konstantinidis KT, Aluru S. 2018. High throughput ANI analysis of 90K prokaryotic genomes reveals clear species boundaries. Nat Commun. 9(1):5114. DOI: 10.1038/s41467-018-07641-9.
10. Zankari E, Hasman H, Cosentino S, Vestergaard M, Rasmussen S, Lund O, Aarestrup FM, Larsen MV. 2012. Identification of acquired antimicrobial resistance genes. J Antimicrob Chemother. 67(11):2640-2644. doi: 10.1093/jac/dks261.
11. Alcock BP, Huynh W, Chalil R, Smith KW, Raphenya AR, Wlodarski MA, Edalatmand A, Petkau A, Syed SA, Tsang KK, Baker SJC, Dave M, McCarthy MC, Mukiri KM, Nasir JA, Golbon B, Imtiaz H, Jiang X, Kaur K, Kwong M, Liang ZC, Niu KC, Shan P, Yang JYJ, Gray KL, Hoad GR, Jia B, Bhando T, Carfrae LA, Farha MA, French S, Gordzevich R, Rachwalski K, Tu MM, Bordeleau E, Dooley D, Griffiths E, Zubyk HL, Brown ED, Maguire F, Beiko RG, Hsiao WWL, Brinkman FSL, Van Domselaar G, McArthur AG. 2023. CARD 2023: expanded curation, support for machine learning, and resistome prediction at the Comprehensive Antibiotic Resistance Database. Nucleic Acids Res. 51(D1):D690-D699. doi: 10.1093/nar/gkac920.
12. Duan Z, Qin J, Liu Y, Li C, Ying C. 2020. Molecular epidemiology and risk factors of Stenotrophomonas maltophilia infections in a Chinese teaching hospital. BMC Microbiol. 20(1):294. doi: 10.1186/s12866-020-01985-3.
13. Vanitshavit V, Charoenlap N, Sallabhan R, Whangsuk W, Bhinija K, Dulyayangkul P, Mongkolsuk S, Vattanaviboon P. 2024. Acquired resistance of Stenotrophomonas maltophilia to antimicrobials induced by herbicide paraquat dichloride. PLoS One. 19(8):e0309525. DOI: 10.1371/journal.pone.0309525.

Table S1. Characteristics of the 60 *S. maltophilia* isolates.

Isolates	MIC (μg/mL) [a]									Biofilm-formation	Swimming motility	Swarming motility	Specific gene				
	Trimethoprim-Sulfamethoxazole (1:19)	Minocycline	Tetracycline	Levofloxacin	Ciprofloxacin	Meropenem	Amikacin	Gentamicin	Colistin				*stmPr1*	*stmPr2*	*smf-1*	*aph(3')IIc*	*smeABC*
all	0.125/2.375-32/608 [b]<br>0.5/9.5<br>4/76	0.5-4<br>1<br>2	8-32<br>16<br>16	1-64<br>6<br>16	2-64<br>8<br>32	64->128<br>>128<br>>128	32->128<br>>128<br>>128	8->128<br>>128<br>>128	32->128<br>64<br>128	strong, 59<br>moderate, 1	strong, 56<br>moderate, 4	negative, 60	+, 35	+, 0	+, 60	+, 60	+, 60
A2	1/19	1	16	**16**	32	>128	>128	128	64	strong	strong	negative	-	-	+	+	+
A3	0.5/9.5	1	16	4	8	128	>128	128	32	strong	strong	negative	-	-	+	+	+
A4	0.25/4.75	0.5	16	2	2	128	>128	128	64	strong	strong	negative	-	-	+	+	+
A5	0.25/4.75	1	16	2	2	>128	>128	128	64	strong	strong	negative	+	-	+	+	+
A6	0.125/2.375	0.5	16	2	2	>128	>128	128	64	strong	strong	negative	+	-	+	+	+
A8	0.25/4.75	2	16	**16**	32	128	>128	64	64	strong	moderate	negative	-	-	+	+	+
B2	0.25/4.75	1	8	4	4	>128	>128	>128	128	strong	strong	negative	+	-	+	+	+
B4	1/19	2	8	**16**	64	>128	>128	128	64	strong	strong	negative	+	-	+	+	+
B5	0.5/9.5	0.5	8	2	4	128	>128	>128	128	strong	strong	negative	+	-	+	+	+
B7	0.5/9.5	1	8	**8**	16	128	>128	>128	64	strong	strong	negative	+	-	+	+	+
C1	0.5/9.5	0.5	8	2	2	64	>128	>128	128	strong	strong	negative	+	-	+	+	+
C2	0.5/9.5	0.5	8	2	4	>128	>128	>128	64	strong	strong	negative	+	-	+	+	+
C3	**8/152**	0.5	8	**16**	32	64	>128	>128	128	strong	strong	negative	+	-	+	+	+
C4	**32/608**	1	8	**16**	32	>128	>128	>128	128	strong	strong	negative	+	-	+	+	+
C5	**8/152**	1	8	**16**	32	128	>128	>128	128	strong	moderate	negative	+	-	+	+	+
C8	**4/76**	2	8	2	4	64	>128	>128	128	strong	strong	negative	-	-	+	+	+
D2	0.5/9.5	2	8	2	2	128	>128	>128	128	strong	strong	negative	-	-	+	+	+
D4	0.25/4.75	1	8	**8**	8	128	>128	128	64	strong	strong	negative	-	-	+	+	+
D5	**8/152**	1	8	**16**	32	128	>128	>128	128	strong	strong	negative	-	-	+	+	+
D6	1/19	2	8	**16**	32	128	>128	>128	128	strong	strong	negative	-	-	+	+	+
D7	**8/152**	2	8	**16**	32	128	>128	>128	64	strong	strong	negative	-	-	+	+	+
D8	0.5/9.5	1	8	1	4	128	>128	64	64	strong	strong	negative	-	-	+	+	+
E1	2/38	1	8	**8**	16	128	>128	>128	64	moderate	strong	negative	-	-	+	+	+
E2	0.5/9.5	**4**	8	**16**	32	>128	>128	>128	>128	strong	strong	negative	+	-	+	+	+
E3	0.25/4.75	0.5	8	2	2	>128	>128	>128	64	strong	strong	negative	-	-	+	+	+
E6	0.25/4.75	2	8	**16**	32	128	>128	>128	128	strong	moderate	negative	+	-	+	+	+
E7	0.25/4.75	2	8	**16**	32	128	>128	>128	128	strong	strong	negative	+	-	+	+	+
E8	0.5/9.5	2	8	**16**	32	128	>128	>128	128	strong	strong	negative	+	-	+	+	+
F5	2/38	2	8	**16**	32	128	>128	128	128	strong	strong	negative	-	-	+	+	+
G1	0.125/2.375	1	16	2	2	>128	>128	128	32	strong	strong	negative	-	-	+	+	+
G2	**4/76**	0.5	8	4	4	>128	>128	>128	32	strong	moderate	negative	-	-	+	+	+
G3	2/38	2	16	**16**	32	>128	64	>128	64	strong	strong	negative	+	-	+	+	+
G4	**16/304**	2	16	**16**	32	>128	32	16	64	strong	strong	negative	-	-	+	+	+
G5	**4/76**	2	16	**16**	16	>128	64	8	64	strong	strong	negative	+	-	+	+	+
G6	**8/152**	2	16	**16**	16	>128	>128	8	64	strong	strong	negative	-	-	+	+	+
G7	**4/76**	2	16	**8**	32	>128	32	>128	64	strong	strong	negative	+	-	+	+	+
G8	**4/76**	2	16	**8**	16	>128	>128	8	64	strong	strong	negative	-	-	+	+	+
H1	0.125/2.375	1	8	4	16	>128	>128	>128	128	strong	strong	negative	+	-	+	+	+
H2	0.25/4.75	0.5	8	2	4	>128	>128	>128	128	strong	strong	negative	+	-	+	+	+
H3	0.125/2.375	0.5	16	2	4	>128	>128	>128	32	strong	strong	negative	+	-	+	+	+
H4	0.25/4.75	0.5	16	2	4	>128	64	>128	32	strong	strong	negative	-	-	+	+	+
H5	0.25/4.75	0.5	16	4	8	128	128	8	64	strong	strong	negative	+	-	+	+	+
H6	0.25/4.75	1	16	4	8	>128	>128	32	64	strong	strong	negative	+	-	+	+	+
H7	0.125/2.375	0.5	8	2	4	>128	>128	>128	128	strong	strong	negative	+	-	+	+	+
H8	0.25/4.75	2	16	**16**	32	>128	>128	>128	64	strong	strong	negative	+	-	+	+	+
I4	2/38	2	16	**16**	32	128	>128	128	64	strong	strong	negative	+	-	+	+	+
I8	0.25/4.75	**4**	32	2	8	>128	>128	>128	64	strong	strong	negative	+	-	+	+	+
J3	0.25/4.75	**4**	32	4	8	>128	>128	>128	64	strong	strong	negative	+	-	+	+	+
J4	**8/152**	1	16	2	4	>128	>128	>128	>128	strong	strong	negative	+	-	+	+	+
J5	**4/76**	0.5	8	2	4	>128	>128	>128	128	strong	strong	negative	+	-	+	+	+
J7	0.125/2.375	1	16	4	16	>128	>128	>128	>128	strong	strong	negative	+	-	+	+	+
K1	0.25/4.75	1	16	4	8	>128	>128	>128	64	strong	strong	negative	+	-	+	+	+
K2	0.25/4.75	0.5	16	4	2	>128	>128	128	64	strong	strong	negative	+	-	+	+	+
K5	0.25/4.75	0.5	16	2	4	>128	>128	128	128	strong	strong	negative	+	-	+	+	+
L2	0.5/9.5	1	16	**8**	8	128	>128	>128	>128	strong	strong	negative	+	-	+	+	+
L3	2/38	1	16	**8**	16	128	>128	>128	>128	strong	strong	negative	-	-	+	+	+
L4	0.5/9.5	0.5	16	2	8	128	>128	>128	128	strong	strong	negative	-	-	+	+	+
L6	0.25/4.75	1	16	**8**	8	>128	>128	>128	>128	strong	strong	negative	-	-	+	+	+
L7	0.5/9.5	1	16	**8**	32	>128	>128	>128	>128	strong	strong	negative	-	-	+	+	+
L8	1/19	1	16	**8**	16	128	>128	>128	128	strong	strong	negative	-	-	+	+	+
ATCC13637 (NBRC14161)	0.5/9.5	0.25	2	0.25	0.25	64	4	4	4	strong	strong	negative	+	+	+	+	+

[a] Bold values indicate resistant isolates according to CLSI breakpoints: trimethoprim-sulfamethoxazole (≧4/76 μg/mL), minocycline (≧4 μg/mL), and levofloxacin (≧8 μg/mL).
Clinical breakpoints for the other antibiotics are not established.

[b] MIC range, MIC50, MIC90 (μg/mL).

Table S2. Characteristics of genome sequence in *S. maltophilia* strains.

Feature	Strain		
	E2	C5	ACYCa.6E [a]
Genome size	4,698,636	4,548,168	4,549,249
CDSs	4,392	4,136	4,292
GC content %	66.5	66.6	66.5
Average Nucleotide Identity % [b]	98.14	98.45	-
Completeness % [c]	99.54	99.64	100
Contamination % [c]	0.34	0.00	0.00
MLST type	ST1482	ST714	ST1347
Prescence of specific gene			
stmpr1 (bprV) [secreted (S8 family) serine protease]	+	+	+
stmpr2 (bprV) [secreted (S8 family) serine protease]	-	-	+
smf-1 [major fimbrial subunit SMF-1] (fimbrial protein)	+	+	+
smlt3773 (*estA*) [outer membrane esterase]	+	+	+
aph3 (*APH*(*3'*)-*IIc*) [aminoglycoside O-phosphotransferase]	+	+	+
aac(*6'*)-*Iz* [aminoglycoside N(6')-acetyltransferase type 1]	+	-	-
blaL1 [metallo-beta-lactamase L1 type 3]	+	+	+
smeABC (*mdtE-ttgB-oprM*) [multidrug efflux RND transporter]	+	+	+
rlmA (*rfbA*) [glucose-1-phosphate thymidylyltransferase]	+	+	+
spgM (*glmM*) [phosphomannomutase]	+	+	+
rpfF (*fadJ*) [faty acid oxidation complex subunit alpha] (crotonase/enoyl-CoA hydratase family protein)	+	± (partial)	+

[a] Reference strain ACYCa.6E (GenBank: CP104290.1).

[b] ANI was calculated using FastANI ACYCa.6E as reference strain.

[c] Completeness and contamination were calculated using CheckM.

Table S3. Primers for detection of virulence, biofilm and antibiotic resistance genes

Name	Gene	Sequence (5´->3’)	Product (bp)	Reference
Stmpr1-F	*Stmpr1*	TGGATGCTTGGTCCCGTAGT	2540	11
Stmpr1-R		CCGTGGTGTCGGCTTCGATCTCT		11
Stmpr2-F	*Stmpr2*	GCCGATTCCGGCATTCACACC	1764	11
Stmpr2-R		GGTCAGGCCCGAGAAGGTGCT		11
Smf-1-F	*Smf-1*	GGAAGGTATGTCCGAGTCCG	674	11
Smf-1-R		GCGGGTACGGCTACGATCAGTT		11
BT8127	*smeABC*	CCCGCATCAACCTCGACTAC	242	12
BT8128		CAGCACCTTTACCTGTGCCT		12
BT8225	*aph*(*3’*)*IIc*	TAATTGCCACCGCCGAAGAA	237	12
BT8226		AGTCATCGGCATCCACCAAC		12
